# Composite Nanostructures for the Production of White Light

**DOI:** 10.3390/molecules29194605

**Published:** 2024-09-27

**Authors:** Giovanni Russo, Francesco Armetta, Tingke Rao, Wangchao Yuan, Vitalii Boiko, Dariusz Hreniak, Cristina Giordano, Maria Luisa Saladino

**Affiliations:** 1 Département de Chimie, Université de Fribourg, PER 10 bu. 402. Ch. du Musée 9, 1700 Fribourg, Switzerland; giovanni.russo@unifr.ch; 2 Dipartimento Scienze e Tecnologie Biologiche, Chimiche e Farmaceutiche—STEBICEF and INSTM UdR—Palermo, Università di Palermo, Viale delle Scienze pad. 17, 90128 Palermo, Italy; francesco.armetta01@unipa.it; 3 Chemistry Department, Queen Mary University of London, Mile End Road, London E1 4NS, UK; t.rao@qmul.ac.uk (T.R.); w.yuan@qmul.ac.uk (W.Y.); 4 Institute of Low Temperature and Structure Research, Polish Academy of Sciences, ul. Okólna 2, 50-422 Wrocław, Poland; v.boiko@intibs.pl (V.B.); d.hreniak@intibs.pl (D.H.)

**Keywords:** white light, gallium indium nitride, YAG:Ce, composite materials, UGR

## Abstract

In this work, two different composite nanostructures, YAG:Ce and Ga_0.9_In_0.1_N, were prepared by the Urea Glass Route method and tested for the production of white light. The first composite was prepared by synthetizing the Ga_0.9_In_0.1_N nanoparticles in the presence of YAG:Ce nanoparticles. The second one was prepared by synthetizing YAG:Ce nanoparticles in the presence of Ga_0.9_In_0.1_N nanoparticles. These systems can be useful for the production of white light. X-ray Diffraction and Transmission and Scanning Electron Microscopies (TEM and SEM) were used to evaluate their structural and morphological properties. Excitation and emission spectra, the quantum yield and colour of the emitted light were acquired to evaluate the optical properties of the systems.

## 1. Introduction

In recent decades, optical devices have been one of the most studied and developed technologies [1]. From the invention of the first lamps through the development of lasers to Light-Emitting Diodes (LEDs), the evolution of performance has been strictly correlated with the properties of the materials used [2]. LEDs led to a revolution in the area of lighting due to their energy efficiency, long lifespan, and environmental benefits. They consume significantly less power compared to traditional incandescent bulbs, reducing electricity costs and greenhouse gas emissions. Additionally, their durability and low maintenance requirements make them ideal for various applications, from residential to industrial settings [3]. White LEDs have rapidly emerged as the next generation of solid-state light sources due to their environmental friendliness and high efficiency and also offer high-quality light that can enhance visual clarity and comfort, improving overall quality of life [4]. Phosphors doped with rare earth ions are extensively used for white light generation [5,6]. The phosphors are generally coated on near-UV/UV/blue LED chips, having the potential for light emission in desirable spectral regions upon the required excitation. The most common type of WLED is produced by combining a gallium indium nitride (Ga_1−x_In_x_N) blue chip [7,8] with a yellow-emitting phosphor, such as YAG:Ce, or *Yttrium Aluminum Garnet* doped with Ce(III) [9]. This combination is useful for improving white LEDs because it combines the high efficiency of GaN blue chips with the broad emission spectrum of the YAG:Ce phosphor. The blue LED chip is covered with a silicone/organic resin embedded with the yellow YAG phosphor [10]. However, the silicone binder encapsulants can easily age and turn yellow owing to the high heat radiation from the high-power white LED, which can degrade the luminous efficacy, shift the chromaticity, and reduce the long-term reliability. Recently, phosphor-in-glass (PiG) film has received extensive attention and achieved fundamental improvements due to its excellent thermal conductivity, high refractive index, and tuneable colour coordination [11]. Polymethylmethacrylate [12,13], polycarbonate [14,15], polyethylene glycol diacrylate (M280), and dipentaerythrityl hexaacrylate (M600) [16] were also investigated as polymers for embedding YAG:Ce. On the other hand, a strong UV emission can be achieved by embedding the GaN-nc powder into a silica-based matrix, influencing its optical properties. This phenomenon can be used to modify the optical properties of free-standing GaN-nc powders [17]. Nanocomposites of GaN nanocrystals with conducting properties show satisfactory white light-emitting properties [18]. GaN-based inorganic/organic structures are employed in several optoelectronic applications [19,20]. GaN ceramics are able to produce an intense broadband laser-induced emission (LIE) in both the NIR and VIS regions [21]. Potentially, ceramics from GaN composites can be used for white light generation [22]. BaAl_2_O_4_-YAG: Ce composite ceramic phosphor is a colour-conversion material used for high-efficiency white LED/LD lighting [23]. Recently, some composite systems based on YAG:Ce and quantum dots were developed to produce more efficient white light. Composite phosphor-based WLEDs using the blends of YAG:Ce and CdSe QDs exhibit an improved luminous efficiency of up to 82 lm/W [24].

In this paper, the development of composite systems composed of Ga_0.9_In_0.1_N and YAG:Ce is reported. The goal of this work is to investigate if the above systems are able to emit warmer white light compared to the YAG:Ce phosphor alone. The mixing of incident blue light with yellow emissions could in fact be enhanced by scattering on nitride nanocrystals. Two composite systems were prepared synthetizing YAG:Ce nanoparticles in the presence of Ga_0.9_In_0.1_N nanoparticles (composite 1, Ga_0.9_In_0.1_N/YAG:Ce) and Ga_0.9_In_0.1_N nanoparticles (NPs) in the presence of YAG:Ce nanoparticles (composite 2, YAG:Ce/Ga_0.9_In_0.1_N).

In both cases, the Urea Glass Route (UGR [25,26]) method was used because it allows the use of non-toxic reagents and short reaction times, and it is a greener method, suitable for scaling up. X-ray Diffraction and Transmission and Scanning Electron Microscopies (TEM and SEM) were used to evaluate the structural and morphological properties. The excitation and emission spectra, quantum yield, and colour of the emitted light were investigated in order to evaluate their potential in the production of white light.

## 2. Results and Discussion

### 2.1. Composite 1 Series—Ga_0.9_In_0.1_N/Ce:YAG

**Figure 1:**
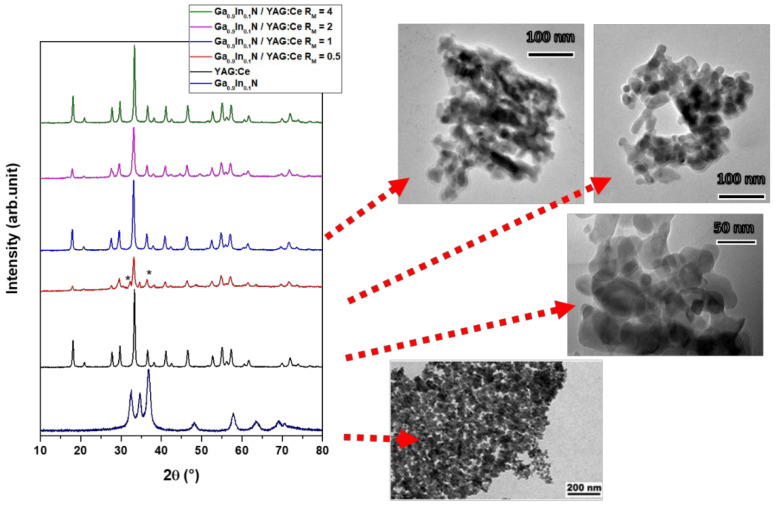
XRD patterns of the Ga_0.9_In_0.1_N/YAG:Ce samples belonging to the composite 2 series varying ***R_M_*_2_**, and of the YAG:Ce and Ga_0.9_In_0.1_N reference samples (ICDD: YAG 00-033-0040, GaN 01-076-0703). The symbol * indicates the gallium nitride phase representative TEM micrographs of each powder.

The XRD patterns and some representative TEM micrographs of the samples belonging to the composite 2 series and of the reference samples Ga_0.9_In_0.1_N and YAG:Ce are reported in Figure 1.

From the XRD patterns, it can be observed that the garnet phase is present in all samples, and only for the sample ***R_M_*_2_** = 0.5 are the peaks of the hexagonal nitride phase observed (indicated with the symbol *). No secondary phases are present. The samples showed clusters of aggregated particles of 200 nm in diameter. These clusters are composed of smaller particles (<50 nm), spheroidal in shape. The elemental mapping for sample ***R_M_*_2_** = 0.5 and 1 showed that the elements that form both the materials are homogeneously covering the whole particle (Figure S1, Supplementary Materials).

**Figure 2:**
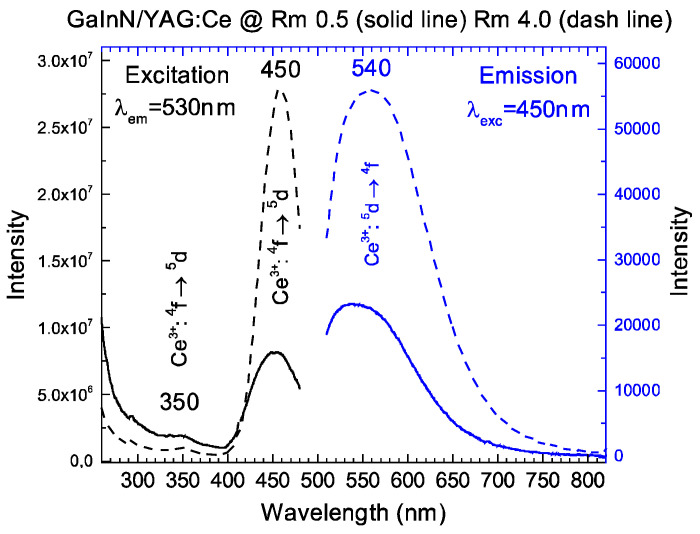
Excitation and emission spectra of Ga_0.9_In_0.1_N/YAG:Ce (R_M2_ = 0.5 and 4).

The excitation spectra of the obtained powders belonging to composite 1 consist of two absorption bands characteristic of Ce^3+^ ions centred at 350 nm and 450 nm assigned to the ^2^F_5/2_→5d^1^ electronic transition. The emission spectra of the obtained materials upon the 450 nm excitation presented reveal the presence of an inhomogeneously broadened emission that consists of two bands related to the 5d→^2^F_5/2_ and 5d→^2^F_7/2_ electronic transition of Ce^3+^ ions. No significant difference can be found in the shape of the emission spectra, confirming the good luminescent properties of the samples. As an example, the excitation (PLE) and emission (PL) spectra of Ga_0.9_In_0.1_N/YAG:Ce (R_M2_ = 0.5 and 4) are reported in Figure 2. Additionally, the emission spectrum is reported in Figure S2 (Supplementary Materials) together with those of YAG:Ce prepared in the same conditions as the comparison. The QY of the best sample is 0.04, lower than the QY of the pure single phase YAG:Ce (0.28).

**Figure 3:**
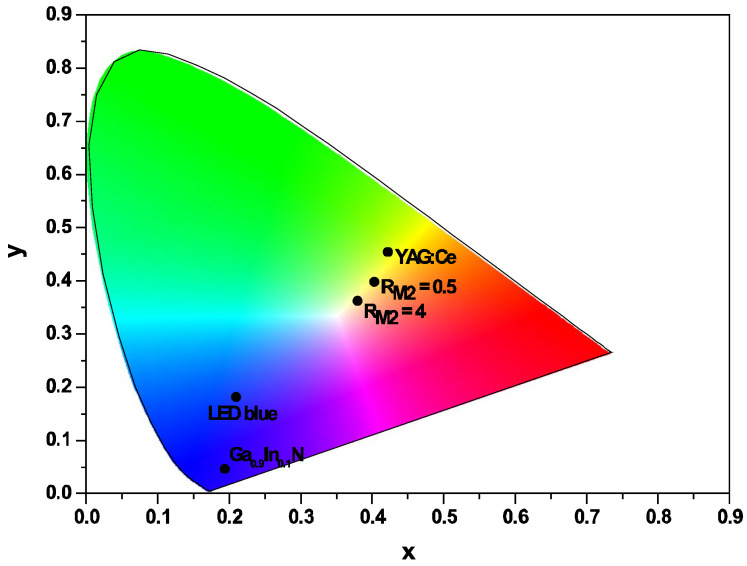
Colour coordinates of blue LED, Ga_0.9_In_0.1_N, YAG:Ce and Ga_0.9_In_0.1_N/YAG:Ce (R_M2_ = 0.5 and 4) samples.

The colour coordinate of the emitted light was evaluated by putting the powders on a blue LED (InGaN, Quantum Light technology, voltage 3.2 V, current 350 mA) in order to verify their potential use for a white light source. The colour of the blue LED and of the Ga_0.9_In_0.1_N powder fall in the blue area. The colour of the YAG:Ce powder and of the composites Ga_0.9_In_0.1_N/YAG:Ce (R_M2_ = 0.5 and 4) fall in the yellow colour area and move to white colour with increasing R_M2_ (Figure 3). This is an indication that these composites can create white light when the R_M2_ values are modulated.

### 2.2. Composite 2 Series—YAG:Ce/Ga_0.9_In_0.1_N

The XRD patterns of the YAG:Ce/Ga_0.9_In_0.1_N series, reported in Figure S3 (Supplementary Materials), show that each sample consists of a crystalline material. In the case of samples ***R_M_*_1_** = 1, 2, and 4, only the garnet phase is present, while increasing the quantity of nitride precursors (samples ***R_M_*_1_** = 6 and 9) led to the presence of the hexagonal nitride phase (peaks between 30° and 40°). However, the excitation and the emission spectra of the YAG:Ce/Ga_0.9_In_0.1_N series show the characteristic band of YAG:Ce [27] but with a very low intensity with respect to the reference YAG:Ce synthesized in the same conditions (Figure S4, Supplementary Materials). The reason for this finding is due to the layer of Ga_0.9_In_0.1_N present on the YAG:Ce particles, as can be observed in the TEM micrographs (Figure S3, Supplementary Materials), where the nanoparticles have a large diameter (100 nm–200 nm) and are found to be agglomerated in bigger clusters. The secondary particles, which are the darker parts of the SEM images, with diameters between 20 nm and 50 nm, agglomerated by forming into larger nanoparticles, jointed by the outer coating. The different contrast indicates a different electron density; YAG:Ce and Ga_0.9_In_0.1_N can be distinguished and the nitride phase seems to be inglobated by the garnet phase. The GaInN content is formed on the surface of the provided YAG:Ce core; as also shown by the elemental mapping for sample ***R_M_*_2_** = 9, a homogeneous distribution of aluminum, yttrium, oxygen, and gallium is present on the investigated particle (Figure S4, Supplementary Materials).

On the other hand, a decrease in both the cell parameters of the nitride lattice is observed, probably due to the diffusion of cations among the crystal structures (Figure S5, Supplementary Materials). In light of these findings, we can assess that the optical properties are not good enough due to the presence of gallium, which modify the garnet phase, and due to the black colour of the powders.

## 3. Materials and Methods

Materials. Urea (Sigma, Tokyo, Japan, 98%), ethanol (Honeywell, Charlotte, NC, USA, 99.98%), Ga(NO_3_)_3_·H_2_O (Aldrich, St. Louis, MO, USA, 99,9%), In(NO_3_)_3_·H_2_O (Aldrich, 99.9%). YCl_3_·6H_2_O (Aldrich, 99.9%), AlCl_3_·6H_2_O (Aldrich, 98%), and CeNO_3_·6H_2_O (Aldrich, 99.99%) were used as received. Solutions were prepared by weight.

Preparation of composites. As starting materials, YAG:Ce and Ga_0.9_In_0.1_N NPs were prepared via the UGR method. YAG:Ce doped with cerium at 1% was prepared following the method reported by Armetta et al. [28]. The Ga_0.9_In_0.1_N NPs were prepared following the method reported by Lei et al. [29]. Two types of composites were prepared: YAG:Ce/Ga_0.9_In_0.1_N and Ga_0.9_In_0.1_N/YAG:Ce.

**Figure 4:**
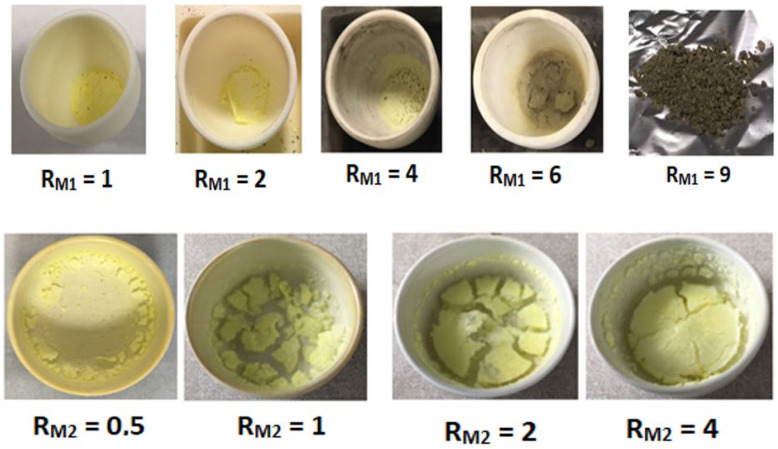
(**up**) YAG:Ce/Ga_0.9_In_0.1_N powders with increasing ***R_M_*_1_**. (**down**) Ga_0.9_In_0.1_N/YAG:Ce powders with increasing ***R_M_*_2_**.

Composite 1 series—Ga_0.9_In_0.1_N/YAG:Ce. Ga_0.9_In_0.1_N NPs were dispersed in ethanol (10 mg/mL) and four solutions of YAG:Ce precursors were prepared. The latter were added to the former and at the end the urea was added to the mixture (**R** = 1, where **R** = (Al^3+^ + Y^3+^ + Ce^3+^/urea). The molar ratio between the components is defined here as YAG:Ce/Ga^3+^, referred to as ***R_M_*_2_** = 0.5, 1, 2, and 4. The dispersions were transferred in crucibles and prepared for the heat treatment: 900 °C, a ramp temperature of 6 °C/min, and a dwelling time of 1 h. The Ga_0.9_In_0.1_N NP dispersion and the Ga_0.9_In_0.1_N NPs + YAG:Ce precursors and urea dispersion are represented in Figure S6 (Supplementary Materials). The obtained samples have a similar yellow colour (Figure 4).

Composite 2 series—YAG:Ce/Ga_0.9_In_0.1_N. Five YAG:Ce NP dispersions in ethanol were prepared at 10 mg/mL. The Ga_0.9_In_0.1_N precursors were thus added to each dispersion at the molar ratio Ga^3+^/YAG:Ce, referred to as ***R_M_*_1_**, equal to 1, 2, 4, 6, and 9, and were sonicated for 10 min. Urea was added (**R** = 5, where **R** = (Ga^3+^ + In^3+^)/Urea) to each dispersion; the systems were thus sonicated and transferred in crucibles for heat treatment. As an example, photos of the YAG:Ce NP dispersion and of the YAG:Ce NP, Ga_0.9_In_0.1_N precursor, and urea dispersion are presented in Figure S1 (Supplementary Materials). The as-prepared dispersions were heat-treated under a nitrogen atmosphere at 800 °C with a temperature ramp of 3 °C/min and a dwelling time of 5 h. The colour of the obtained powders varied from yellow to brown depending on the ***R_M_*_1_**, different from the colour of both the YAG:Ce and the Ga_0.9_In_0.1_N pure single phase (Figure 1).

Characterization Techniques. XRD patterns were acquired by a Philips PW 1050/39 diffractometer in the Bragg–Brentano geometry using Ni-filtered Cu Kα radiation (λ = 1.54056 Å) in the 2θ range 5–90° with a step of 0.05° and a step time of 5 sec. The X-ray generator’s power levels were 40 kV and 30 mA, and the resolution of the instrument (divergent and antiscatter slits of 0.5°) was determined using R-SiO_2_ and R-Al_2_O_3_ standards free from the effect of reduced crystallite size and lattice defects. The phase identification was performed by using the X’pert HighScore^®^ Software v5.1. In order to obtain information about phase composition, the cell parameters, and the crystalline size of the phases, the XRD patterns were analyzed according to the Rietveld method [30] by using the MAUD software v.2.999 [31]. The *TEM micrographs* were acquired using a JOEL 2010 at a voltage of 200kV. The samples were first ground to a fine powder, then suspended in ethanol. One drop of this suspension was put on a holey carbon-coated copper grid of 300 mesh and left to air-dry. The SEM *micrographs* were acquired using a FEI inspect F instrument. The samples were loaded on carbon-coated stubs and coated by sputtering with a Au/Pd alloy before the observation.

*Photoluminescence emission (PL)* and *excitation (PLE) spectra* as well as external *quantum yield (QY)* were measured using the FLS980 Fluorescence Spectrometer from Edinburgh Instruments Ltd. (Livingston, UK). As an excitation source, a 450 W Xenon lamp (for PL and PLE) was used. Both the excitation and emission 300 mm focal length monochromators were in Czerny–Turner configuration. The excitation arm was supplied with a holographic grating of 1800 lines/mm and blazed at 300 nm, while the emission spectra were supplied with a ruled grating of 1800 lines/mm blazed at 500 nm. The spectral resolution was 0.1 nm. An R928P side window photomultiplier tube from Hamamatsu was used as a detector. For the *QY* measurements, an integrating sphere (SM4 from Edinburg Instruments) with a diameter of 30 cm was additionally used.

An Ocean Optics reflectance spectrophotometer equipped with a DH-mini light source and a USB 2000 + XR1 detector, operating in the spectral range 200–1100 nm, was used to calculate the *colour coordinates of the light* generated by lighting the samples with a blue LED (GaInN, Quantum Light Technology, Vilnius, Lithuania, 3.2 V and 350 mA). Data were acquired by setting a CIE D65 standard illuminant and a CIE 10° standard colorimetric observer. The CIE 1931 XYZ colour space was used as a reference system.

## 4. Conclusions

The possible development of white light from a composite system made by YAG:Ce and Ga_0.9_In_0.1_N is reported here. The results show that the colour of the obtained powders can be influenced by the preparation steps. In fact, if the Ga_0.9_In_0.1_N particles are synthesized in the presence of YAG:Ce (YAG:Ce/Ga_0.9_In_0.1_N, composite 2,), the colour of the sample is affected by the Ga_0.9_In_0.1_N amount, changing to a black colour, and the optical properties are not enough good for white light production. This is probably due to the change in the cell parameters of the garnet structure, which increases due to the diffusion of cations in the lattice. The composite 1 system Ga_0.9_In_0.1_N/YAG:Ce, prepared by synthesizing the YAG:Ce particles in the presence of Ga_0.9_In_0.1_N and possessing a yellow colour, shows the emission of white light at proper ***R_M_*_1_**.

This work demonstrates that the composite Ga_0.9_In_0.1_N/YAG:Ce system has the potential to be used in devices for the emission of warm white light via a simple, un-expensive, and scalable methodology. More interesting is the observed enhancement of the yellow emissions due to the scattering on nitride nanocrystals.

## Data Availability

The datasets generated during and/or analyzed during the current study are available from the corresponding author on reasonable request.

## Supplementary Materials

The following supporting information can be downloaded at: https://www.mdpi.com/article/10.3390/molecules29194605/s1, Figure S1. Elemental mapping of sample RM_2_ = 0.5 and 1.; Figure S2. Excitation (PLE) and photoemission spectra (PE) of YAG:Ce/Ga0.9In0.1N (RM1 = 9). Spectra of YAG:Ce prepared in the same conditions is reported as comparison. Figure S3. XRD patterns of the YAG:Ce/Ga0.9In0.1N samples belonging to the Composite 1 series varying RM_1_ and of YAG:Ce and Ga0.9In0.1N reference samples. The symbol * indicates the GaN phase. (ICDD: YAG 00-033-0040, GaN 01-076-0703). Figure S4. Elemental mapping of sample RM_2_ = 9. Figure S5. Cell parameter a of garnet phase in the YAG:Ce/Ga0.9In0.1N series, varying RM_1_. For RM1 = 0 indicates a of the reference YAG:Ce. Figure S6. Ethanolic dispersions of YAG:Ce NPs; YAG:Ce NPs + Ga0.9In0.1N precursors and urea (composite 1), and of Ga0.9In0.1N NPs and Ga0.9In0.1N NPs + YAG:Ce precursors and urea (composite 2).
