# Supplementary material for: Composite Nanostructures for the Production of White Light
Source: Molecules. 2024 Sep 27;29(19):4605. doi: 10.3390/molecules29194605 (PMC11477575; doi:10.3390/molecules29194605)
Supplement: Supplementary file 1 [file molecules-29-04605-s001.zip › molecules-3153716-supplementary.pdf]

Support Information

Composite Nanostructures for the Production of White Light

Giovanni Russo [1], Francesco Armetta [2], Tingke Rao [3], Wangchao Yuan [3], Vitalii Boiko [4], Dariusz Hreniak [4], Cristina Giordano [3,*] and Maria Luisa Saladino [2,*]

[1] Département de Chimie, Université de Fribourg, PER 10 bu. 402. Ch. du Musée 9, 1700 Fribourg, Switzerland; giovanni.russo@unifr.ch
[2] Dipartimento Scienze e Tecnologie Biologiche, Chimiche e Farmaceutiche—STEBICEF and INSTM UdR—Palermo, Università di Palermo, Viale delle Scienze pad. 17, 90128 Palermo, Italy; francesco.armetta01@unipa.it
[3] Chemistry Department, Queen Mary University of London, Mile End Road, London E1 4NS, UK; t.rao@qmul.ac.uk (T.R.); w.yuan@qmul.ac.uk (W.Y.)
[4] Institute of Low Temperature and Structure Research, Polish Academy of Sciences, ul. Okólna 2, 50-422 Wrocław, Poland; v.boiko@intibs.pl (V.B.); d.hreniak@intibs.pl (D.H.)
* Correspondence: c.giordano@qmul.ac.uk (C.G.); marialuisa.saladino@unipa.it (M.L.S.)

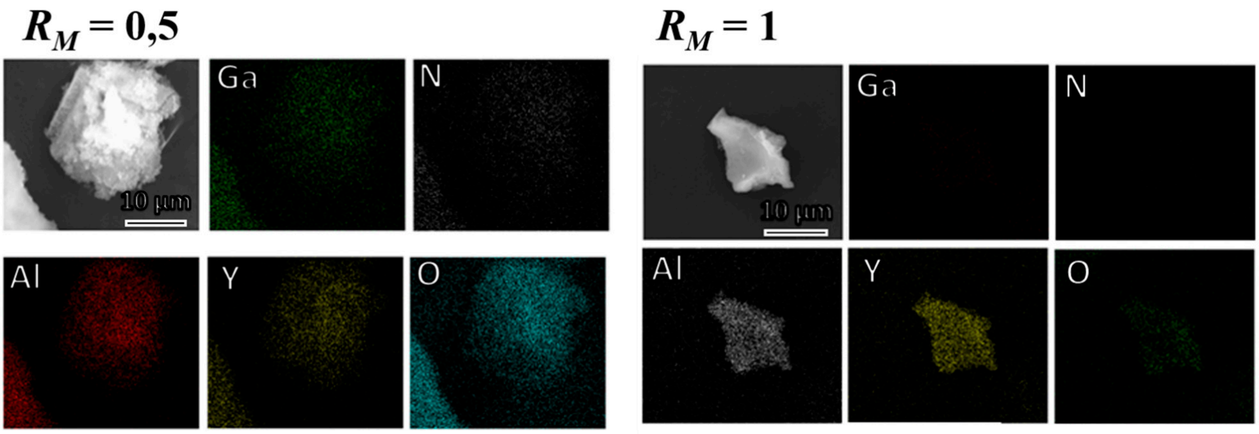

Figure S1. Elemental mapping of sample $\boldsymbol{R}_{M2}$ = 0.5 and 1.

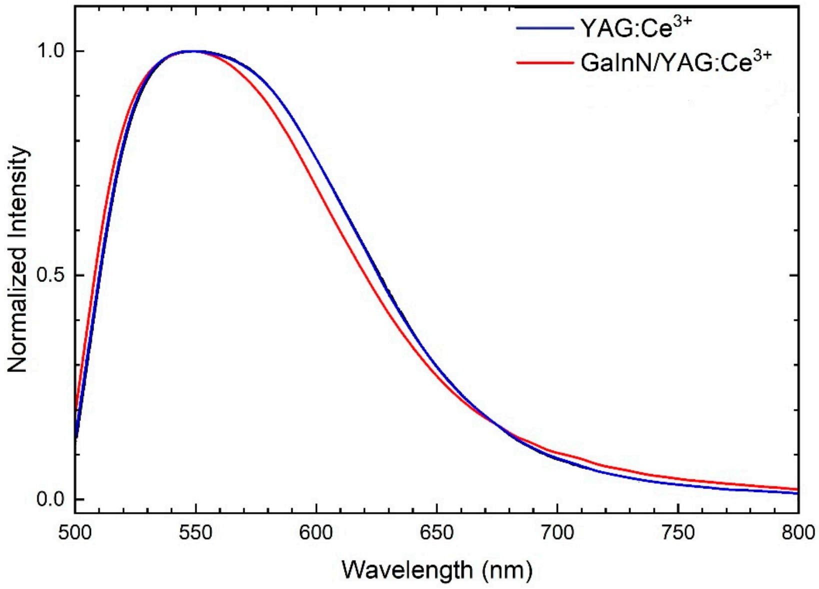

Figure S2. Excitation (PLE) and photoemission spectra (PE) of YAG:Ce / $Ga_{0.9}In_{0.1}N$ (R_{M1}= 9). Spectra of YAG:Ce prepared in the same conditions is reported as comparison.

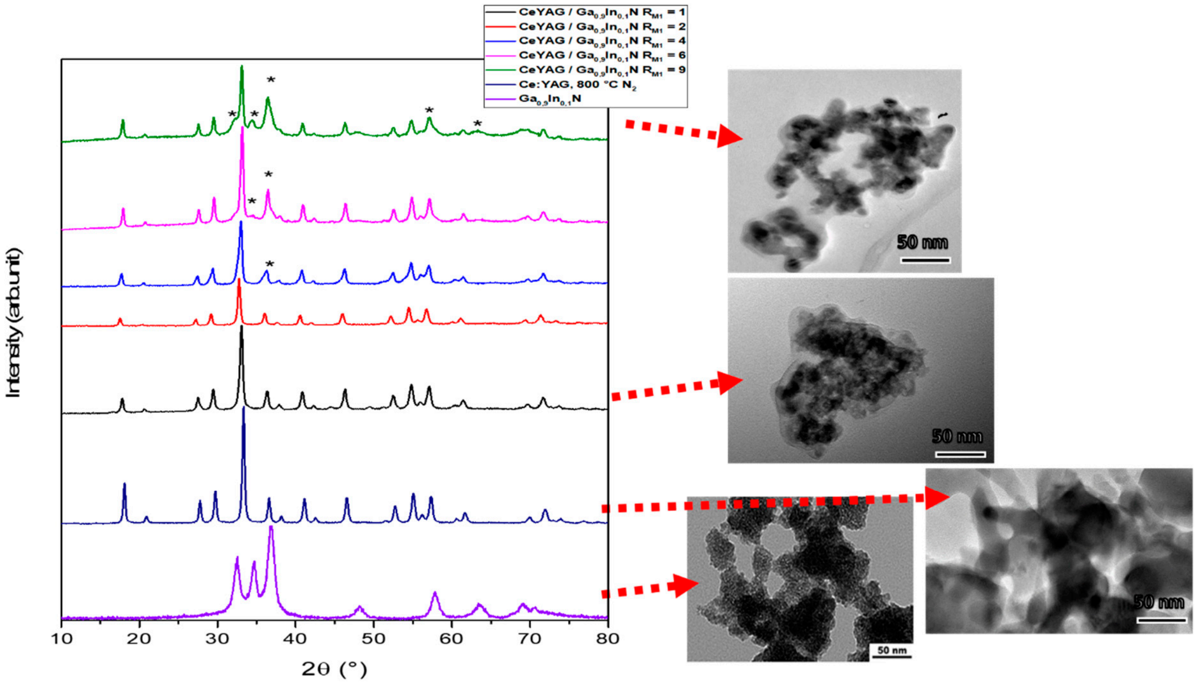

Figure S3. XRD patterns of the YAG:Ce/$Ga_{0.9}In_{0.1}N$ samples belonging to the Composite 1 series varying R_{M1} and of YAG:Ce and $Ga_{0.9}In_{0.1}N$ reference samples. The symbol * indicates the GaN phase. (ICDD: YAG 00-033-0040, GaN 01-076-0703).

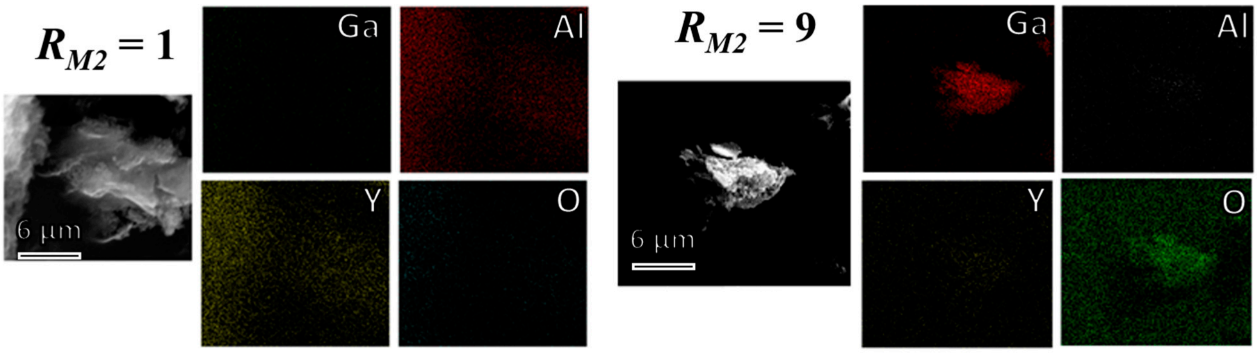

Figure S4. Elemental mapping of sample R_{M2} = 9.

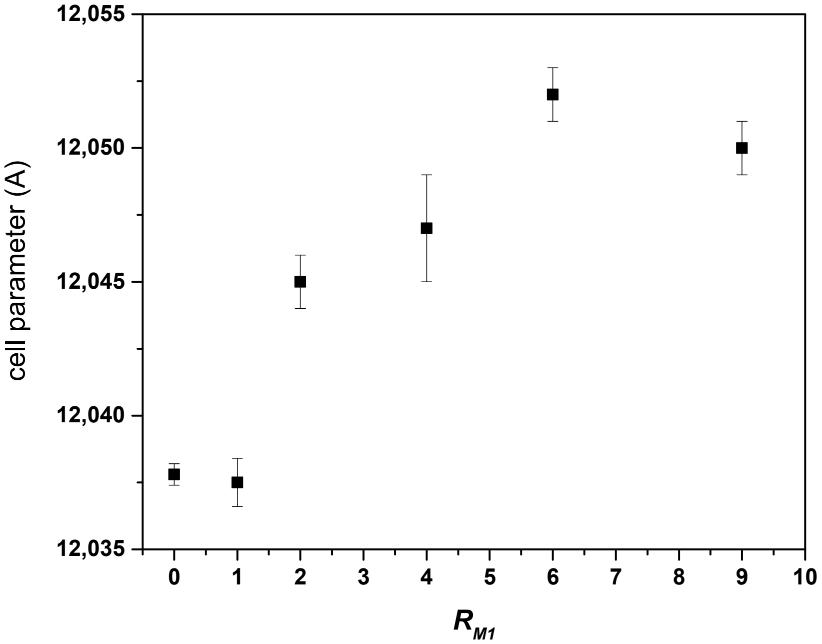

Figure S5. Cell parameter *a* of garnet phase in the YAG:Ce/ $Ga_{0.9}In_{0.1}N$ series, varying R_{M1}. For R_{M1} = 0 indicates *a* of the reference YAG:Ce.

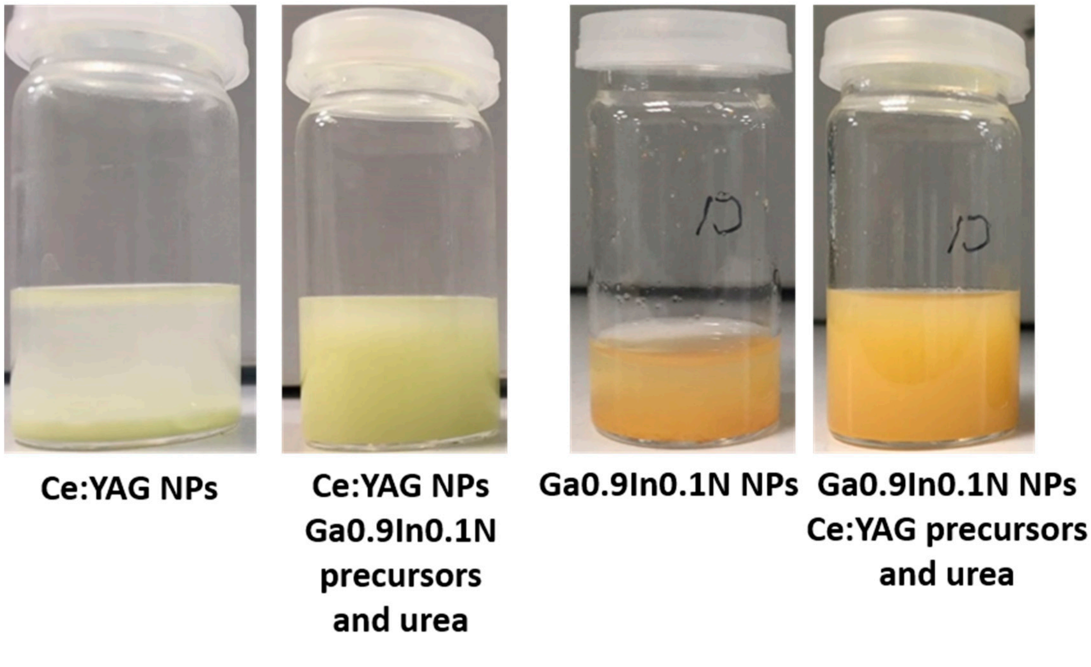

Figure S6. Ethanolic dispersions of YAG:Ce NPs; YAG:Ce NPs + $Ga_{0.9}In_{0.1}N$ precursors and urea (composite 1), and of $Ga_{0.9}In_{0.1}N$ NPs and $Ga_{0.9}In_{0.1}N$ NPs + YAG:Ce precursors and urea (composite 2).
